# Profile of Mortal Injuries in Vehicular Accidents: An Autopsy-Based Study

**DOI:** 10.7759/cureus.61425

**Published:** 2024-05-31

**Authors:** Ashok Jiwane, Shailesh Raut, Harshal R Thube, Mandar Sane, Manish Shrigiriwar

**Affiliations:** 1 Forensic Medicine, All India Institute of Medical Sciences (AIIMS) Nagpur, Nagpur, IND

**Keywords:** motorcycle rider, vehicular accident, autopsy, thoracoabdominal injuries, head injury

## Abstract

Background

It is crucial to analyze the trends of fatal injuries among pedestrians, passengers, motorcycle riders, and drivers of three- and four-wheelers in traffic accidents.

Objective

To ascertain the trend of fatal injuries to the head, chest, and abdomen across different victim categories in vehicular accidents.

Materials and methods

An autopsy-based prospective study was carried out in the mortuary of a rural tertiary care hospital. A total of 108 fatal cases of vehicular accidents were taken into consideration. All natural and unnatural deaths, other than those stemming from vehicle crashes, were excluded from this study; only the victims of fatal vehicular accidents were included.

Results

Males outnumbered female victims by 8.8:1. The age range of 41-60 years was the most affected (38.9%). The greatest number of victims (17, or 15.8%) were male motorcycle riders in the range of 21-40 years. Most vehicular mishaps (61; 56.5%) occurred during the evening. The most frequent injury pattern reported was head injuries (53.4%).

Conclusions

Motorcycle riders constituted the most severely injured victim category in a vehicular accident. Most mishaps occurred in the dark because of inadequate lighting or bad road conditions in rural areas. Furthermore, the most frequently occurring type of injury was an injury to the head, which may be an outcome of riders' lack of compliance with the mandatory helmet-use policy.

## Introduction

An accident is “an unfortunate event that happens unexpectedly and unintentionally, typically resulting in damage or injury” [1]. A vehicle accident is “an event which takes place on a way or street open to public traffic; resulting in one or more than one individual being injured or killed, where at least one moving vehicle is involved" [2]. Road traffic accident (RTA)-related injuries are becoming a notifiable issue for public health worldwide due to society's rising motorization and globalization. In December 2018, the WHO released the Report on Global Road Safety Status, which found that 1.35 million people pass away from traffic-related deaths every year. RTA injuries are currently the major cause of demise for those between the ages of 5 and 29 [3]. Vehicular accident injuries are predicted to become the fifth most frequent cause of death by 2030 [4]. Although only 60% of vehicles are owned by people in low- and middle-income countries, these nations account for 90% of all traffic fatalities globally [5]. Trauma primarily affects the head, chest, abdomen, extremities, and perineum in fatal vehicular crashes. Motorcycle accidents that result in death most often include head injuries [6]. In Indian cities, walking alongside roads has always been unsafe, but over the past few years, the risk has increased due to encroachment onto walkways. Based on data from traffic accidents in 2018, West Bengal led the list with 2618 pedestrian deaths, followed by Maharashtra with 2515 deaths [7]. This research is being conducted in Maharashtra's countryside where people are compelled to travel on poorly designed and maintained village roads. In comparison to four-wheelers, there are more motorcyclists involved in fatal vehicular accidents.

## Materials and methods

This prospective descriptive study was undertaken between November 1, 2017, and October 1, 2019, in the morgue of Swami Ramanand Teerth Rural Government Medical College, a tertiary medical facility. A total of 967 autopsies were performed during the study period. Of those, 290 (30%) were deaths due to natural causes and 677 (70%) were deaths due to unnatural causes. A total of 108 (11.2%) out of the 677 unnatural deaths were caused by vehicular accidents. All natural and unnatural deaths, other than those stemming from vehicle accidents, were excluded from this study, with only the victims of fatal vehicular accidents included. Informed consent was acquired from the next of kin of the deceased. Records of every case were obtained from admission documents, families, witnesses, and investigative officers. The current study categorizes victims of fatal vehicular accidents as PED (Pedestrian), PR (Pillion Rider), MCR (Motorcycle Rider), 3W&4WD (Three-Wheeler Driver and Four-Wheeler Driver), PASS (Passengers), and MVD (Multi-axle Vehicle Driver). Additionally, a six-hour time frame was used to determine the proportion of victims affected in each victim category. The accident spots were chosen within a 50-kilometer radius of the rural medical center. Parameters such as age, gender, incidence period, and injury pattern were studied.

Statistical method of analysis

Using the Microsoft Excel tool (Microsoft Corporation, Redmond, WA, USA), observational data from the proforma was entered into the master chart. IBM SPSS (v28.0.1; IBM Corp, Armonk, NY, USA) was subsequently utilized to obtain the results through statistical methods and essential filter tools.

## Results

Demographic distribution of victim categories (N=108)

There was an 8.8:1 ratio of male-to-female victims, with 97 (89.9%) male victims as compared to 11 female victims (10.1%). The age range of 41-60 years was the most frequently involved, with 42 (38.9%) victims. Most of the victims in the age group of 21-40 were male motorcycle riders, with 17 (15.8%) fatalities. Within the age range of 41-60 years, there were 13 pedestrians (12%). The most recorded victim category among females was found to be pillion riders, with 5 (4.6%) deaths. See Table 1 for the distribution.

**Table 1:** Demographic distribution of victim categories (N = 108)

Category of victims		Age in years				Total
		< 20	21–40	41–60	≥60	
Pedestrian	Male	4	7	12	7	30 (27.8%)
	Female	2	0	1	0	03 (2.8%)
	Total	6 (5.5%)	7 (6.5%)	13 (12%)	7 (6.4%)	33 (30.6%)
Pillion rider	Male	0	1	5	1	07 (6.5%)
	Female	0	2	2	1	05 (4.6%)
	Total	0	3 (2.7%)	7 (6.5%)	2 (1.8%)	12 (11.1%)
Motorcycle rider	Male	8	17	12	1	38 (35.2%)
	Female	0	0	0	0	00
	Total	8 (7.4%)	17 (15.8%)	12 (11.1%)	1 (0.9%)	38 (35.2%)
3-wheeler driver and 4-wheeler driver	Male	1	3	1	0	05 (4.6%)
	Female	0	0	0	0	00
	Total	1 (0.9%)	3 (2.75)	1 (0.9%)	0	05 (4.6%)
Passengers	Male	1	5	8	0	14 (13%)
	Female	1	2	0	0	03 (2.8%)
	Total	2 (1.8%)	7 (6.5%)	8 (7.4%)	0	17 (15.7%)
Multi-axle vehicle driver	Male	0	2	1	0	03 (2.8%)
	Female	0	0	0	0	00
	Total	0	2 (1.8%)	1 (0.9%)	0	03 (2.8%)
Total	Male	14	35	39	9	97 (89.9%)
	Female	3	4	3	1	11 (10.1%)
	Total	17 (15.7%)	39 (36.1%)	42 (38.9%)	10 (9.3%)	108 (100%)

Victim categories affected at different accident timings (N = 108)

For accident timing, a six-hourly time frame was considered. The time between 06.00 PM and 11.59 PM was the most perilous period, with 61 (56.5%) incidents. The victims most frequently involved in accidents in this period were motorcyclists, with 28 fatalities (25.9%), followed by pedestrians with 17 (16.7%). Table 2 lists the victim categories affected at different accident timings.

**Table 2:** Victim category affected in different accident timing (N = 108) PED: Pedestrian, PR: Pillion Rider, MCR: Motorcycle Rider, 3W&4WD: 3-Wheeler and 4-Wheeler Driver, PASS: Passengers, MVD: Multi-axle Vehicle Driver

Time of Accident	PED (%)	PR (%)	MCR (%)	3W & 4WD (%)	PASS (%)	MVD (%)	Total (%)
12.00 AM – 05.59 AM	3 (2.8%)	0	2 (1.8%)	1 (0.9%)	3 (2.8%)	1 (0.9%)	10(9.3%)
06.00 AM – 11.59 AM	5 (4.7%)	3 (2.8%)	2 (1.8%)	1 (0.9%)	3 (2.8%)	1 (0.9%)	15(13.9%)
12.00 PM – 05.59 PM	7 (6.5%)	4 (3.7%)	6 (5.5%)	0	5 (4.6%)	0	22(20.4%)
06.00 PM – 11.59 PM	17 (16.7%)	5 (4.6%)	28 (25.9%)	3 (2.8%)	6 (5.6%)	1 (0.9%)	61(56.5%)
Total	33 (30.6%)	12 (11.1%)	38 (35.2%)	05 (4.6%)	17 (15.7%)	03 (2.8%)	108 (100%)

Different body regions affected in the victim’s category (N = 189)

Our observation during the autopsy was that individuals often had multiple body regions impacted. This explains the N value in Table 3, which reveals the most compromised body region was the head, with 101 (53.4%) injuries. Among motorcycle riders, the most commonly injured body region was the head at 19%, followed by the thorax (11.1%) and abdomen (6.9%).

**Table 3:** Different body regions affected in the victim’s category (N = 189)

Category of Victim	Head (%)	Thorax (%)	Abdomen (%)	Total (%)
Pedestrian	31 (16.4%)	17 (9%)	7 (3.7%)	55 (29.1%)
Pillion rider	9 (4.8%)	4 (2.1%)	2 (1.1%)	15 (7.9%)
Motorcycle rider	36 (19%)	21 (11.1%)	13 (6.9%)	70 (37%)
3W and 4W drivers	5 (2.6%)	5 (2.6%)	1 (0.5%)	11 (5.8%)
Passengers	17 (9%)	11 (5.8%)	5 (2.6%)	33 (17.5%)
Multi-axle vehicle driver	3 (1.6%)	2 (1.1%)	0	5 (2.6%)
Total	101 (53.4%)	60 (31.7%)	28 (14.8%)	189 (100%)

Thoracic involvement in the victim’s category (N = 60)

As noted in Table 4, thoracic injuries were reported in 60 victims (31.7%), making this the second most common after head injuries. There were significant cases where multiple injuries were observed in a single person, resulting in overlapping injury sites. In the thoracic region, the most significant injuries observed were rib fractures, as seen in 46 cases (76.7%), followed by lung contusions in 36 (60%). Heart laceration was infrequent, with only 3 instances (5%).

**Table 4:** Thoracic involvement in the victim’s category (N=60) LC: Lung Contusion, LL: Lung Laceration, HL: Heart Laceration, FR: Fracture of Rib; 3W: Three Wheeler, 4W: Four Wheeler

Category of Victim	LC (%)	LL (%)	HL (%)	FR (%)
Pedestrian	11 (18.3%)	10 (16.7%)	1 (1.7%)	13 (21.7%)
Pillion rider	4 (6.7%)	1 (1.7%)	0	4 (6.7%)
Motorcycle rider	7 (11.7%)	7 (11.7%)	1 (1.7%)	14 (23.3%)
3W and 4W drivers	4 (6.7%)	2 (3.3%)	0	3 (5%)
Passengers	9 (15%)	8 (13.3%)	1 (1.7%)	11 (18.3%)
Multi-axle vehicle driver	1 (1.7%)	1 (1.7%)	0	1 (1.7%)
Total	36 (60%)	29 (48.3%)	3 (5%)	46 (76.7%)

Abdominal involvement in the victim’s category (N = 28)

Injury to the abdomen was present in 28 victims (14.8%). The most common abdominal injuries were kidney contusions with 14 (50%) incidences and liver lacerations with 13 (46.4%). Motorcycle riders reported the most liver lacerations with 8 cases (28.6%), whereas passengers showed the highest incidence of kidney contusion with 5 (17.8%). No injury was reported to the abdominal region among multi-axle vehicle drivers. Table 5 lists the abdominal involvement in the victim's category.

**Table 5:** Abdominal involvement in the victim’s category (N = 28) LiC: Liver Contusion, LiL: Liver Laceration, SL: Spleen Laceration, KC: Kidney Laceration, FP: Fractured Pelvis

Category of Victim	LiC (%)	LiL (%)	SL (%)	KC (%)	KL (%)	FP (%)
Pedestrian	2 (7.1%)	1 (3.6%)	4 (14.3%)	3 (10.7%)	0	1 (3.6%)
Pillion rider	1 (3.6%)	0	0	2 (7.1%)	0	0
Motorcycle rider	2 (7.1%)	8 (28.6%)	3 (10.7%)	3 (10.7%)	0	1 (3.6%)
3W and 4W drivers	0	0	0	1 (3.6%)	1 (3.6%)	0
Passengers	1 (3.6%)	4 (14.3%)	2 (7.1%)	5 (17.8%)	1 (3.6%)	2 (7.1%)
Multi-axle vehicle driver	0	0	0	0	0	0
Total	6 (21.4%)	13 (46.4%)	9 (32.1%)	14 (50%)	2 (7.1%)	4 (14.3%)

## Discussion

As populations continue to surge and urban areas expand rapidly, the strain on transportation infrastructure, especially roads, intensifies in developing nations. Consequently, the rising number of fatalities from road traffic accidents is becoming a concerning trend in these regions.

In the current study, 11.2% of deaths from vehicular accidents were reported to the mortuary. Males are more commonly involved in accidents, with the male-to-female ratio being 8.8:1. Male preponderance in vehicular accidents is probably due to the greater likelihood of them traveling to and from work each day, as compared to females who conventionally perform household activities. The studies of Singh et al. and Anand et al. observe the same result [2,8].

Victims more frequently came from the age range of 41-60 years, with 42 cases (38.9%). Within the age range of 41-60 years, there were 13 (12%) pedestrians. Mandal et al. had the same findings, but Sahu et al. and Reddy N et al. report that the age group from 21 to 40 years is the one most commonly involved in fatal accidents, citing figures of 20.80% and 50%, respectively [9-11]. Some of the probable causes include the expansion of shops and illegal buildings, which creates a hurdle for elderly people who wish to stroll on the walkways. Vehicle users' reckless driving exacerbates this issue. Additional factors could include the physical characteristics of those in this age group, weakened vision, and slower reflexes.

This study has found that the majority of RTAs (61; 56.5%) occur in the hours between 06.00 PM and 11.59 PM. The victim categories most frequently impacted in this period were motorcyclists, with 28 fatalities (25.9%), followed by pedestrians with 17 (16.7%). Reddy N et al., Gupta et al., and Katageri et al. observe the same findings [11-13]. Evenings are a time when traffic jams occur on the roadways because people are rushing to get home from work. Most of these roads also have insufficient lighting, especially in the surrounding suburban and rural areas and on the edges of cities.

The most compromised body region was the head with 101 incidences (53.4%). Among motorcycle riders, the most commonly injured body region was the head at 19%, followed by the thorax at 11.1%, and the abdomen at 6.9%. At only 1.6%, head injuries were rare among multi-axle vehicle drivers. Pillion riders had the lowest reported incidence of thoracic injuries (1.1%). Gupta et al. find that the most common head injury is seen in pedestrians (84%) [12]. However, our observation is contraindicated by Reddy N et al. who found that thoracic injury is the most common (73%) [11]. Our finding correlates with studies of Lamb et al., Kaur et al., and Guntheti et al. with regard to the head being the most vulnerable body component in traffic accidents, especially among motorcycle riders [14-16].

According to research, rib fractures were observed in 76.7% of victims, and lung contusions in 60%. Rib fractures were most often seen in motorcyclists (23.3%) and then in pedestrians (21.7%). The prevalence of lung contusion (18.3%) and lung laceration (16.7%) was highest among pedestrians. Singh et al. and Numan et al. argue the same [2,17]. The heart, lungs, and other vital organs are located within the thoracic cavity. An individual's health is seriously in danger if they suffer trauma in this area. When motorcyclists and pedestrians collide, the impact can cause serious injuries to the chest. There may be little external evidence of an injury but there may be significant internal harm such as multiple rib fractures, lung contusions, and thoracic organ lacerations.

In the current study, the most frequent abdominal injuries were kidney contusion (50%), liver laceration (46.4%), and laceration of the spleen (32.1%). However, these observations are opposed by Numan et al. [17] and Singh et al. [2] who found that liver laceration is the most common. In this study, contusion to the spleen was not reported. The abdomen is sometimes called the "magic box" of the body because it can sustain injuries from a range of acute shocks, including vehicular accidents. As the abdominal wall is elastic, internal organ crushing does not result in visible exterior damage. The consistency, mobility, degree of organ distension, kind of force, impact location, and level of resistance provided by the abdominal wall in any given circumstance all influence the extent of damage after trauma.

Limitations of the study

This study does not cover urban vehicular accidents. The wide variety of crash patterns observed in urban areas cannot be studied here, as this study is limited to rural regions; hence, our sample size is limited. A small sample size may decrease the study's statistical power and limit its applicability.

Suggestions

The lack of sidewalks for elderly people to walk on owing to shop encroachment, unlawful construction, etc., is often cited as the cause of traffic accidents. This issue is made worse by careless driving on the part of car users. Road traffic laws, such as those that strictly enforce the wearing of safety helmets, the creation of pedestrian-friendly pathways, the designation of separate lanes for light and heavy vehicles, and the strict enforcement of traffic rules and regulations, are necessary to reduce the number of fatal traffic accidents.

## Conclusions

In the current research, 108 deaths resulting from vehicular accidents were studied. There were more male deaths than female. Most of the deceased belonged to the 41-60 age range. Notably, 56.5% of fatal vehicle accidents occurred in the evening, specifically from 6.00 PM to 11.59 PM. The most frequent injury pattern in this study was a head injury which was often sustained by motorcycle riders. Motorcycle riders sustained head, thoracic, and abdominal injuries as 19%, 11.1%, and 6.9% of injuries, respectively. Fractured ribs were a persistent finding among thoracic injuries. Kidney contusion was regularly observed among abdominal injuries.
